# Primary Melanoma of the Urinary Bladder: Clinical, Histopathologic, and Comprehensive Molecular Analysis of a Rare Tumor

**DOI:** 10.1177/10668969241283486

**Published:** 2024-10-03

**Authors:** Neslihan Kayraklioglu, Emily Chan, Boris Bastian, Steven R. Long

**Affiliations:** 1 Department of Pathology, 8785 University of California, San Francisco, CA, USA

**Keywords:** mucosal melanoma, urinary bladder, molecular analysis

## Abstract

Primary melanoma of the urinary bladder is extremely rare and generally has a poor prognosis. The histopathological diagnosis can be challenging as tumors can be unpigmented and of varying morphology. Here we report a rare example of primary urinary bladder melanoma with clinical, imaging, gross anatomical, histopathologic, immunohistochemical, and molecular findings to illustrate the utility of an integrated approach in establishing the diagnosis and guide therapy. A comprehensive, integrated approach, including molecular studies, may be helpful in further establishing an accurate diagnosis and informing therapies of this rare but poorly behaved entity.

## Background

Mucosal melanomas are rare tumors, consisting of about 1% of all melanomas.^1^ They arise from melanocytes that reside in mucosal tissues and are mostly seen in oral, nasal, genital, and rectal mucosae. In contrast to cutaneous melanoma, ultraviolet radiation is not involved in its pathogenesis. The molecular landscape of mucosal melanoma is characterized by a low point mutation burden, with high numbers of copy number and structural variants and mutations in mitogen-activated protein kinase (MAPK) pathway genes such as *NRAS, BRAF, NF1,* and *KIT*, as well as *SF3B1*, *TP53*, *SPRED1, ATRX, HLA-A, CDH8,* and *CTNNB1*.^2–5^ Overall, mucosal melanomas have a significantly worse outcome compared to cutaneous melanomas with 5-year survival rates ranging from 20% to 25%.^6,7^

The genitourinary tract is a rare location for mucosal melanomas, the urethra being the most common site.^8^ There are <50 examples of primary urinary bladder melanomas reported in the literature.^8–10^ Tumors usually present with macroscopic hematuria and dysuria.^10^ Cystoscopy is the primary modality for recognition and usually shows a dark-pigmented mass with varying dimensions. Primary bladder melanomas can have variable histomorphology and can be misdiagnosed as urothelial carcinoma, sarcoma, or metastatic cutaneous melanoma.^11^ Melanocytic markers such as SOX10, Melan-A, and HMB-45 are usually positive. Molecular alterations specific to primary urinary bladder melanomas are rarely reported in the literature and are mainly composed of a few case reports and small case series with limited molecular analysis.^8,11^ More comprehensive genomic studies are needed to understand the pathogenesis of primary urinary bladder melanomas and identify possible targeted treatment modalities.

Here we report a primary bladder melanoma, describing clinical, morphological, and immunohistochemical findings and molecular analysis.

## Case Presentation

**Figure 1.**
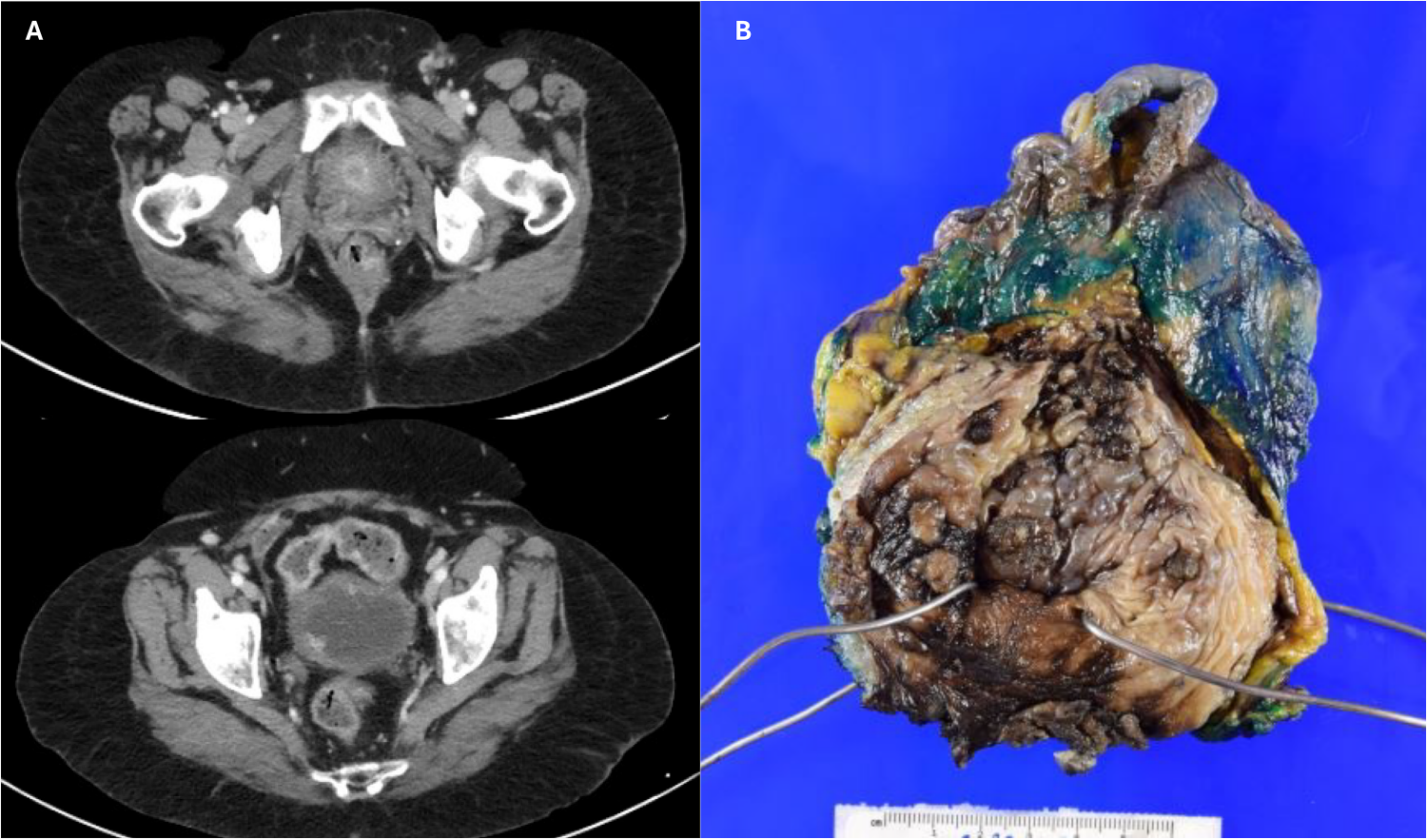
Radiographic and macroscopic findings. (A) PET/CT showing multifocal hyperenhancing lesions in the bladder, the largest at the anterior bladder neck (upper panel) and an additional lesion near the right posterior bladder base (lower panel), (B) gross image of urinary bladder with multiple foci of brown–gray, friable, nodular masses.

PET: positron emission tomography; CT: computed tomography.

A 70-year-old woman presented with complaints of gross hematuria and was evaluated with computed tomography (CT) urogram, which revealed a focal linear filling defect in the proximal right ureter with a heterogeneous mass in the inferior border. Positron emission tomography/CT showed multifocal hyperenhancing lesions in the bladder, the largest at the anterior bladder neck and orifice with likely extension into the urethra (measuring 1.9 × 1.5 cm) and an additional lesion near the right posterior bladder base (1.4 cm in maximum diameter) (Figure 1A). No definite evidence of hypermetabolic metastatic disease outside of the bladder was seen. Subsequent dermatological examination revealed no suspicious skin lesions or sites of a non-urologic primary.

The patient underwent transurethral resection followed by radical cystectomy with urethrectomy and pelvic lymph node dissections.

### Macroscopic Findings

The cystectomy specimen showed multiple foci of brown–gray, friable, nodular masses (approximately 8 × 7 cm in aggregate area) throughout the trigone, dome, anterior, right lateral, and posterior wall with scattered small foci in the left lateral and posterior wall; overall covering ∼75% of the bladder mucosa (Figure 1B). The maximum depth of the masses measured 0.6 cm, with possible invasion into the muscularis propria. The lesions extended to the urethral margin and were within 0.1 cm of the anterior perivesical soft tissue margin.

A separately submitted urethral meatus resection specimen was firm with no distinct masses.

### Microscopic Findings

**Figure 2.**
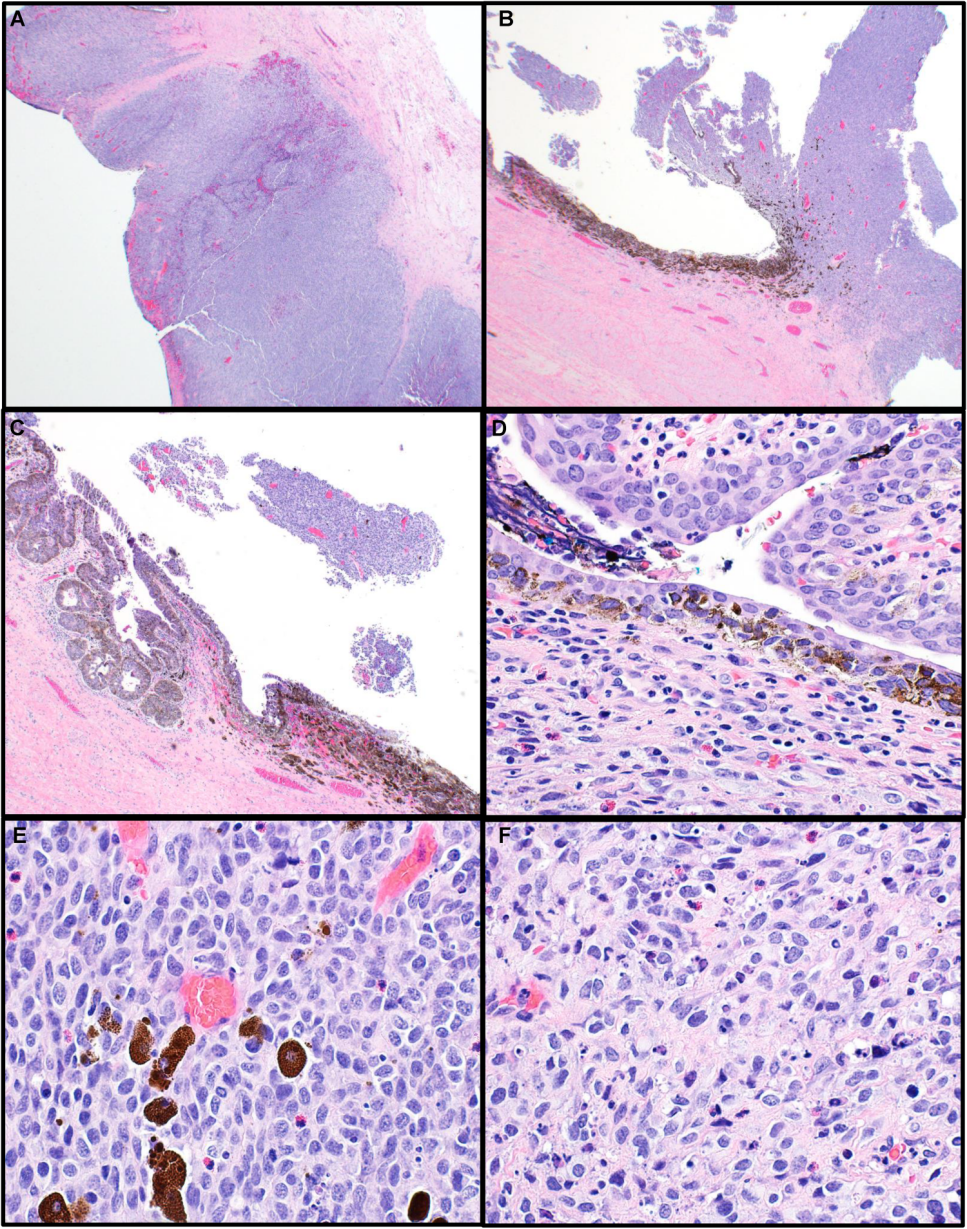
Morphological findings. Hematoxylin and eosin (H&E)-stained sections showed sheets of cells forming intramural nodules (A), exophytic masses (B), and flat lesions demonstrating a pagetoid spread of the neoplastic cells throughout the urothelium (C, D). (E, F) The neoplastic cells have spindled to epithelioid morphology with abundant mitotic figures, apoptotic debris, and an inflammatory cell infiltrate. Dark brown pigment was occasionally present in the atypical cells (D) and the intermixed macrophages (E) (original magnifications A–C 20×; D–F: 400×).

Hematoxylin and eosin (H&E) stained sections of the mass showed sheets of cells forming intramural nodules (Figure 2A), exophytic masses protruding toward the lumen (Figure 2B) as well as flat lesions demonstrating pagetoid spread of neoplastic cells throughout the urothelium (Figures 2C and 2D). The neoplastic cells were slightly dyscohesive, spindled to epithelioid, with moderate amounts of amphophilic cytoplasm, irregular and hyperchromatic nuclei with abundant mitotic figures, apoptotic debris, and an inflammatory cell infiltrate (Figures 2E and 2F). The dark brown pigment was occasionally present in neoplastic cells and intermixed macrophages (Figures 2B, 2C, and 2E).

**Figure 3.**
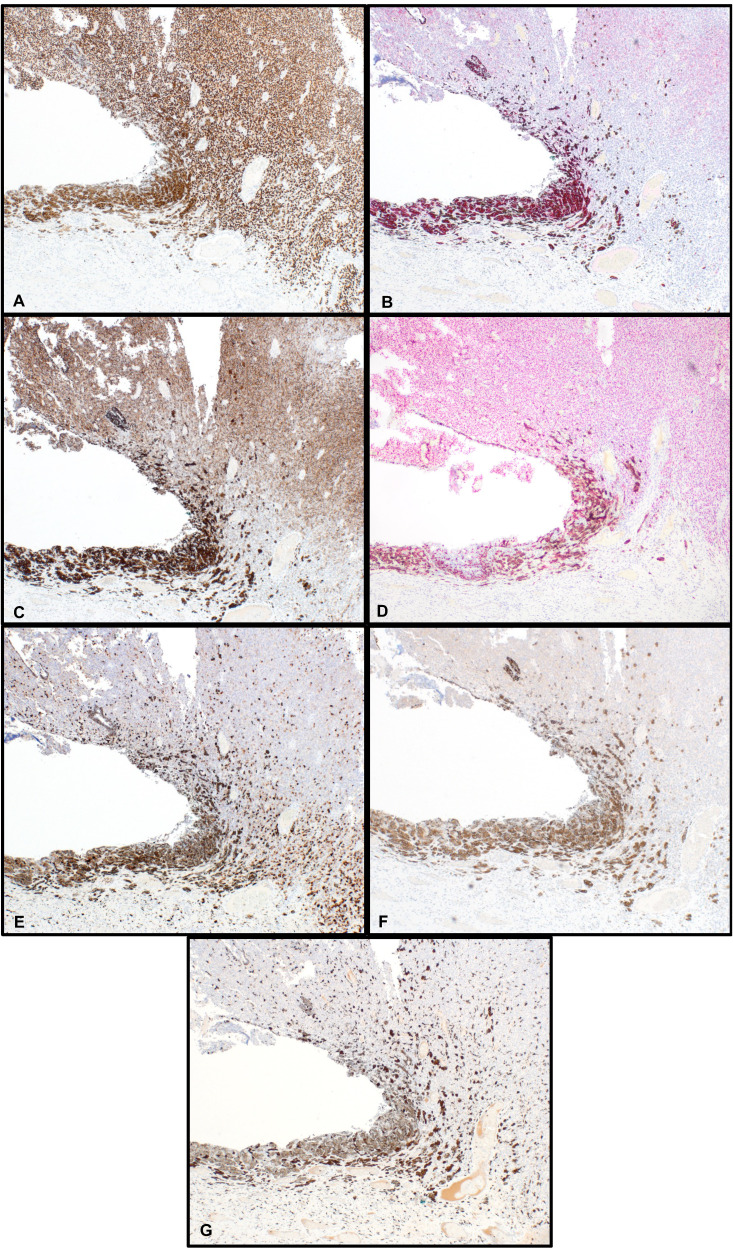
Immunohistochemical findings. Neoplastic cells are diffusely positive for SOX10 (A), Melan-A (B), HMB-45 (C), preferentially expressed antigen in melanoma (PRAME) (D), patchy positive for S100 (E) and negative for p63 (F). CD163 (G) highlights intermixed pigmented macrophages (original magnification: 40×).

Immunohistochemical studies showed diffusely positive SOX10 (Figure 3A), Melan-A (Figure 3B), HMB-45 (Figure 3C), preferentially expressed antigen in melanoma (PRAME) (Figure 3D) and patchy S100 (Figure 3E) immunoreactivity and were negative for p63 (Figure 3F) and GATA3, supporting the diagnosis of melanoma. A CD163 stain highlighted admixed pigmented macrophages (melanophages) (Figure 3G). The tumor invaded into lamina propria but no definite muscularis propria invasion was identified. The separately submitted urethral meatus resection specimen showed a small subepithelial nodule of melanoma and melanoma in situ extending to the mucosal resection margin. No tumor was identified in the submitted lymph nodes.

### Molecular Studies

Hybrid-capture-based next-generation sequencing was performed at the University of California San Francisco Clinical Cancer Genomics Laboratory, using an assay targeting the coding regions of 479 cancer genes, as well as select introns of 47 genes frequently involved in rearrangements (UCSF500 Cancer Gene Panel). Analysis showed numerous copy number changes and multiple focused amplifications including a high-level amplification of the *KIT* locus with *PDGFRA* and *KDR* coamplifications and amplifications of *EP300, SOX10,* and *CRKL* genes. Single nucleotide variant analysis showed a frameshift mutation in *NF1*. No ultraviolet signature was identified.

### Clinical Outcome

On follow-up, adjuvant immunotherapy was recommended although ultimately not pursued. The patient was followed up elsewhere with systemic therapy status unknown to us. She later developed widely metastatic disease and died within 43 months of initial diagnosis.

## Discussion

Primary mucosal melanoma of the urinary bladder is an uncommon malignancy often presenting with hematuria and cystoscopy findings of a pigmented lesion/mass which are not entirely specific and could be seen in a variety of benign or malignant conditions. At the benign end of the differential diagnosis spectrum is the melanosis of the urinary bladder, which is another rare entity of unknown pathogenesis. Although melanin pigment is present, melanosis is not thought to be composed of melanocytes. The morphologic findings of pigmented bland urothelium with no invasive/mass-like lesion along with negative melanocytic markers are helpful clues to differentiate this entity from melanomas.^12,13^

Like melanomas at other anatomical locations, primary melanomas of the urinary bladder can display varying morphology including epithelioid, clear, spindled, and rhabdoid cells with nested, diffuse, and fascicular growth patterns.^8,11^ Histological diagnosis can be challenging especially when melanin pigment is absent. The variable morphology brings up the differential of high-grade or poorly differentiated urothelial carcinoma, sarcomas, and metastatic cutaneous melanoma, all of which occur more frequently in the urinary bladder than primary mucosal melanoma. Immunohistochemistry can assist in the diagnosis, particularly of amelanotic tumors.

Cutaneous melanoma metastatic to the bladder occurs in 18% of patients who die from metastatic melanoma; therefore, careful physical examination is required to assess this possibility.^14^ The following criteria were suggested for considering melanoma a primary lesion in urinary bladder: (1) no history of a previous cutaneous lesion; (2) no evidence of regressed cutaneous malignant melanoma; (3) no evidence of other visceral primary melanoma; (4) the pattern of recurrence should be consistent with the findings in the region of initial malignant melanoma; and (5) margins of bladder lesion should contain atypical melanocytes similar to those seen in the periphery of primary mucous membrane lesions.^15,16^ BRAF V600E mutations are uncommon in mucosal melanoma and immunohistochemistry for the BRAF V600E, although not entirely specific, can be a helpful tool for distinguishing primary mucosal melanoma from metastases originating from cutaneous melanoma.

Assessment of these criteria relies significantly on a complete and accurate clinical history with a thorough radiological and physical examination. It is possible that a portion of the primary bladder melanoma tumors presented in the literature, especially those with widespread metastasis, could represent metastases from undetected or regressed cutaneous melanomas. In the current patient, dermatological examination showed no foci of cutaneous melanocytic lesions and there were no additional foci detected by radiological imaging. While a regressed cutaneous melanoma cannot be entirely excluded, the presence of an in situ portion at the margin of the tumor was in keeping with primary mucosal melanoma. We cannot be entirely sure whether the tumor originated from the urinary bladder or the proximal urethra as it involved both locations at presentation, although the majority of the mass was centered in the urinary bladder favoring the urinary bladder as the most likely origin.

Molecular studies are helpful in differentiating metastatic versus primary mucosal melanomas. In contrast to the vast majority of cutaneous melanomas, mucosal melanomas do not contain characteristic ultraviolet-induced C > T nucleotide transition signatures and have low mutational burden and a greater number of structural chromosomal variants.^17^ Whole exome sequencing studies in a cohort of various mucosal melanoma subtypes revealed frequent mutations in MAPK pathway genes such as *NRAS, BRAF, NF1,* and *KIT* as well as *SF3B1, TP53, SPRED1, ATRX, HLA-A, CDH8,* and *CTNNB1* genes.^3,4^ Mutational profile may differ among the mucosal melanomas depending on their anatomical location, for example, mucosal melanomas of the urethra show a higher frequency of *TP53* mutations compared to vulvar/vaginal melanomas.^18^ There are rare case reports and a small case series of primary melanomas of the urinary bladder with limited molecular analysis, revealing alterations in *BRAF, FGFR1,* and *ERBB2* genes.^8,11^ BRAF V600E mutations are rare in mucosal melanomas, although it was reported in 3 out of 5 patients in a case series of primary melanomas of the urinary bladder^5,11,19–21^ (Table 1). Without additional evidence, this raises the possibility that these may represent metastases rather than primary mucosal melanoma. Mucosal melanomas are genetically characterized by highly rearranged genomes with numerous copy number changes, including multiple focused amplifications with a low mutation burden, as seen in our patient.^3,5,22^ Somatic mutations in our patient included a frameshift mutation in *NF1* as is common in mucosal melanoma.^21^ In addition, there were amplifications of the loci encoding *EP300, SOX10,* and *CRKL,* all genes recurrently amplified in mucosal melanoma *NF1* inactivating mutations are common in mucosal melanomas and frequently co-occur with *KIT* activation.^23,24^ In this tumor, there is a focused high-level amplification of the *KIT* locus. While *PDGFRA* and *KDR* are co-amplified, *KIT* is considered the driver oncogene at this locus as it is recurrently mutated in mucosal melanomas.^21^ The frequent co-occurrence of *KIT* activating mutations or amplification with *NF1* or *SPRED1* inactivation has led to the suggestion of using combination inhibitors of *KIT* and *MEK* for tumors driven by these alterations.^24,25^

**Table 1.** Primary melanomas of the urinary bladder with molecular alterations reported in the literature.

Study	Patients/tumor location	Molecular method	Molecular alterations	Treatment	Outcome (F/U)
Karabulut et al.^11^	52 M, left lateral wall	*BRAF* PCR	*BRAF* mutation	Radical cystoprostatectomy, LND	NED (60)
	63 F, apex		*BRAF* mutation	Radical cystoprostatectomy, LND, vemurafenib	NED (12)
	76 F, unknown		*BRAF* mutation	TUR, vemurafenib	AWD (15)
	54 M, unknown		Negative for *BRAF* mutation	TUR	Deceased (4)
	70 M, unknown		Negative for *BRAF* mutation	None	Deceased (32)
Acikalin et al.^8^	62 M, anterior wall	Next-generation sequencing	*ERBB2* and *FGFR1* copy number decrease	Unknown	Deceased (22)
Current study	70 F, anterior bladder neck, right posterior bladder base and urethra	Next-generation sequencing	*KIT*, *PDGFRA*, *KDR*, *EP300*, *SOX19*, *CRKL* amplifications, *NF1* mutation	Radical cystectomy, LND	Deceased (43)

Abbreviations: M, male; F, female; LND, lymph node dissection; TUR, transurethral resection; NED, no evidence of disease; AWD, alive with disease; F/U, follow up (months); PCR: polymerase chain reaction.

Mucosal melanomas are generally detected at advanced stages and have a poor prognosis.^26^ Unlike cutaneous melanoma, mucosal melanomas don’t respond well to immunotherapy and to date, only limited actionable driver mutations have been identified. There is no established staging guideline and no standardized treatment for primary urinary bladder melanomas. Treatment options include transurethral resection or radical cystectomy followed by chemotherapy, radiotherapy, or immunotherapy. Molecular studies can help explore possible targeted treatment options such as *KIT* and *MEK* inhibitor therapy in this situation.^25^ More studies are needed to establish reliable staging and treatment guidelines for primary urinary bladder melanoma. With a further understanding of the genomic landscape of primary urinary bladder melanomas, actionable mutations for targeted treatment strategies could be identified.

## References

[1] Mikkelsen LH Larsen AC von Buchwald C Drzewiecki KT Prause JU Heegaard S . Mucosal malignant melanoma—a clinical, oncological, pathological and genetic survey. APMIS. 2016;124(6):475-486. doi:10.1111/apm.12529 27004972

[2] Hintzsche JD Gorden NT Amato CM , et al. Whole-exome sequencing identifies recurrent SF3B1 R625 mutation and comutation of NF1 and KIT in mucosal melanoma. Melanoma Res. 2017;27(3):189-199. doi:10.1097/CMR.0000000000000345 28296713 PMC5470740

[3] Newell F Kong Y Wilmott JS , et al. Whole-genome landscape of mucosal melanoma reveals diverse drivers and therapeutic targets. Nat Commun. 2019;10(1):3163. doi:10.1038/s41467-019-11107-x 31320640 PMC6639323

[4] Zhou R Shi C Tao W , et al. Analysis of mucosal melanoma whole-genome landscapes reveals clinically relevant genomic aberrations. Clin Cancer Res. 2019;25(12):3548-3560. doi:10.1158/1078-0432.CCR-18-3442 30782616

[5] Curtin JA Fridlyand J Kageshita T , et al. Distinct sets of genetic alterations in melanoma. N Engl J Med. 2005;353(20):2135-2147. doi:10.1056/NEJMoa050092 16291983

[6] Kuk D Shoushtari AN Barker CA , et al. Prognosis of mucosal, uveal, acral, nonacral cutaneous, and unknown primary melanoma from the time of first metastasis. Oncologist. 2016;21(7):848-854. doi:10.1634/theoncologist.2015-0522 27286787 PMC4943393

[7] Bishop KD Olszewski AJ . Epidemiology and survival outcomes of ocular and mucosal melanomas: a population-based analysis. Int J Cancer. 2014;134(12):2961-2971. doi:10.1002/ijc.28625 24272143

[8] Acikalin A Bagir E Karim S Bisgin A Izol V Erdogan S . Primary melanoma of the urinary tract; clinicopathologic and molecular review of a case series. Pathol Res Pract. 2020;216(9):153095. doi:10.1016/j.prp.2020.153095 32825962

[9] Dai JD He B Liu ZH Shi M Shen PF . Primary melanoma of the bladder: case report and review of the literature. World J Surg Oncol. 2022;20(1):287. doi:10.1186/s12957-022-02753-5 36071438 PMC9454232

[10] Venyo AK . Melanoma of the urinary bladder: a review of the literature. Surg Res Pract. 2014;2014:1-13. doi:10.1155/2014/605802 PMC4208590 25374957

[11] Karabulut YY Erdogan S Sayar H Ergen A Ertoy Baydar D . Primary malignant melanoma of the urinary bladder: clinical, morphological, and molecular analysis of five cases. Melanoma Res. 2016;26(6):616-624. doi:10.1097/CMR.0000000000000300 27603550

[12] Di Fiore F Pone D Domizio S , et al. Melanosis of the bladder: a rare endoscopic and histopathological finding. Urol Int. 2007;78(2):185-187. doi:10.1159/000098082 17293664

[13] Sawalem K Mehdi MZ Ansari AH Ramadan A . Melanosis of bladder: a rare entity. Urol Case Rep. 2019;27:100916. doi:10.1016/j.eucr.2019.100916 31687352 PMC6819767

[14] Dasgupta T Brasfield R . Metastatic melanoma. A clinicopathological study. Cancer. 1964;17:1323-1339. doi:10.1002/1097-0142(196410)17:10<1323::aid-cncr2820171015>3.0.co;2-n 14236766

[15] Ainsworth AM Clark WH Mastrangelo M Conger KB . Primary malignant melanoma of the urinary bladder. Cancer. 1976;37(4):1928-1936. doi:10.1002/1097-0142(197604)37:4<1928::aid-cncr2820370444>3.0.co;2-w 1260695

[16] Stein BS Kendall AR . Malignant melanoma of the genitourinary tract. J Urol. 1984;132(5):859-868. doi:10.1016/s0022-5347(17)49927-7 6387180

[17] Furney SJ Turajlic S Stamp G , et al. Genome sequencing of mucosal melanomas reveals that they are driven by distinct mechanisms from cutaneous melanoma. J Pathol. 2013;230(3):261-269. doi:10.1002/path.4204 23620124

[18] Mano R Hoeh B DiNatale RG , et al. Urethral melanoma—clinical, pathological and molecular characteristics. Bladder Cancer. 2022;8(3):291-301. doi:10.3233/BLC-211633 36277327 PMC9536426

[19] Zarei S Voss JS Jin L , et al. Mutational profile in vulvar, vaginal, and urethral melanomas: review of 37 cases with focus on primary tumor site. Int J Gynecol Pathol. 2020;39(6):587-594. doi:10.1097/PGP.0000000000000636 31567539

[20] Schindler K Schicher N Kunstfeld R , et al. A rare case of primary rhabdoid melanoma of the urinary bladder treated with ipilimumab, an anti-CTLA 4 monoclonal antibody. Melanoma Res. 2012;22(4):320-325. doi:10.1097/CMR.0b013e32835566c0 22713795

[21] Curtin JA Busam K Pinkel D Bastian BC . Somatic activation of KIT in distinct subtypes of melanoma. J Clin Oncol. 2006;24(26):4340-4346. doi:10.1200/JCO.2006.06.2984 16908931

[22] Hayward NK Wilmott JS Waddell N , et al. Whole-genome landscapes of major melanoma subtypes. Nature. 2017;545(7653):175-180. doi:10.1038/nature22071 28467829

[23] Cosgarea I Ugurel S Sucker A , et al. Targeted next generation sequencing of mucosal melanomas identifies frequent NF1 and RAS mutations. Oncotarget. 2017;8(25):40683-40692. doi:10.18632/oncotarget.16542 28380455 PMC5522195

[24] Ablain J Xu M Rothschild H , et al. Human tumor genomics and zebrafish modeling identify SPRED1 loss as a driver of mucosal melanoma. Science. 2018;362(6418):1055-1060. doi:10.1126/science.aau6509 30385465 PMC6475924

[25] Subbiah V Baik C Kirkwood JM . Clinical development of BRAF plus MEK inhibitor combinations. Trends Cancer. 2020;6(9):797-810. doi:10.1016/j.trecan.2020.05.009 32540454

[26] Cazzato G Colagrande A Cimmino A , et al. Urological melanoma: a comprehensive review of a rare subclass of mucosal melanoma with emphasis on differential diagnosis and therapeutic approaches. Cancers (Basel). 2021;13(17):4424. doi:10.3390/cancers13174424 34503234 PMC8431506

